# Supplementary material for: Integrated Transcriptome and sRNAome Analysis Reveals the Molecular Mechanisms of Piriformospora indica-Mediated Resistance to Fusarium Wilt in Banana
Source: Int J Mol Sci. 2024 Nov 20;25(22):12446. doi: 10.3390/ijms252212446 (PMC11595150; doi:10.3390/ijms252212446)
Supplement: Supplementary file 1 [file ijms-25-12446-s001.zip › Fig.S4.pdf]

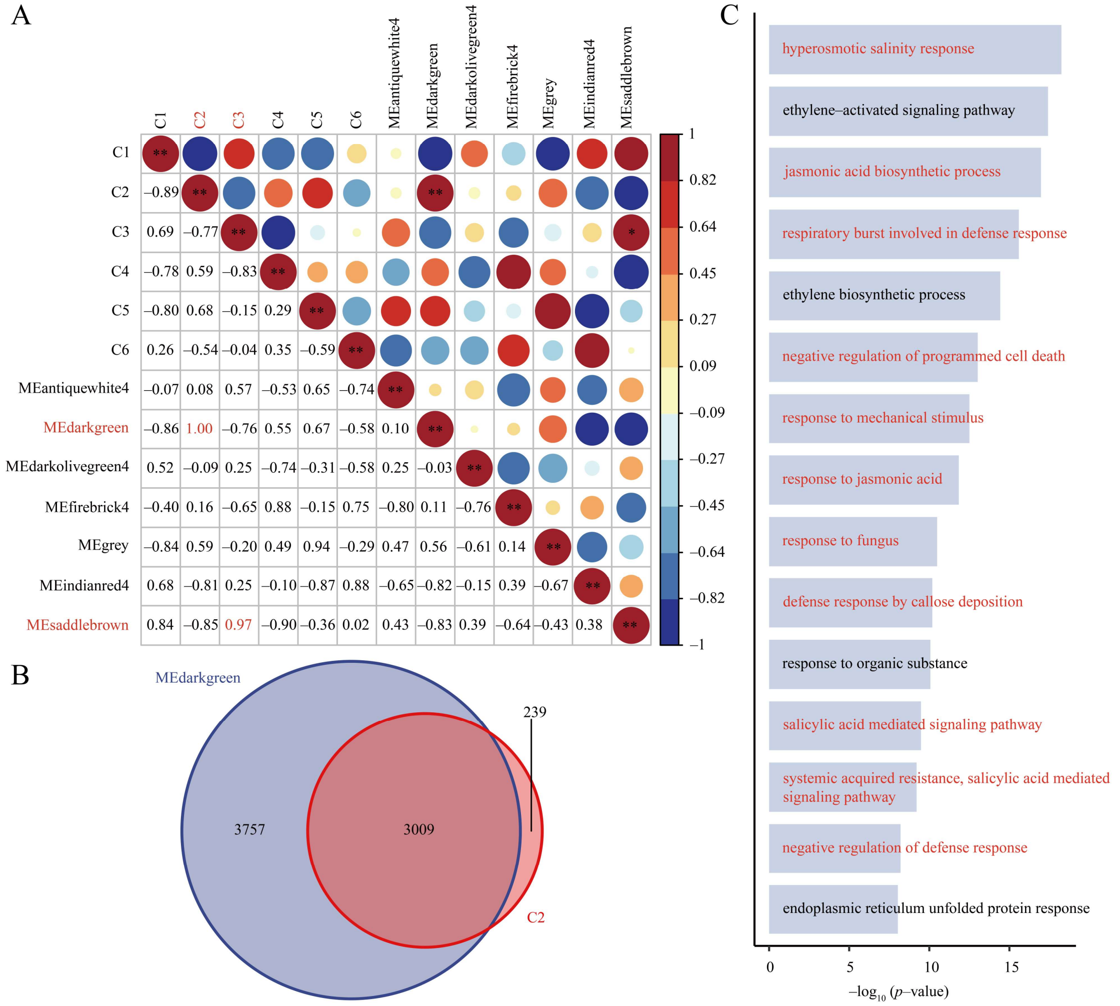

Figure S4. Association analysis between hierarchical clustering groups and WGCNA modules. (A) Heatmap of Pearson correlation between the 13 modules. Highlight MEdarkgreen, MEsaddlebrown, and the Pearson correlation between them in red font. (B)Venn diagram representing the gene intersection between hierarchical cluster 2 and the MEdarkgreen module, totaling 3009 genes. (C) GO analysis of 3009 which only focus on the biological process (BP) enrichment results. The red content is the same as pathways marked in the C5 module in Fig. 3B.
