# Supplementary figures and images for: Integrated Transcriptome and sRNAome Analysis Reveals the Molecular Mechanisms of Piriformospora indica-Mediated Resistance to Fusarium Wilt in Banana
Source: Int J Mol Sci. 2024 Nov 20;25(22):12446. doi: 10.3390/ijms252212446 (PMC11595150; doi:10.3390/ijms252212446)

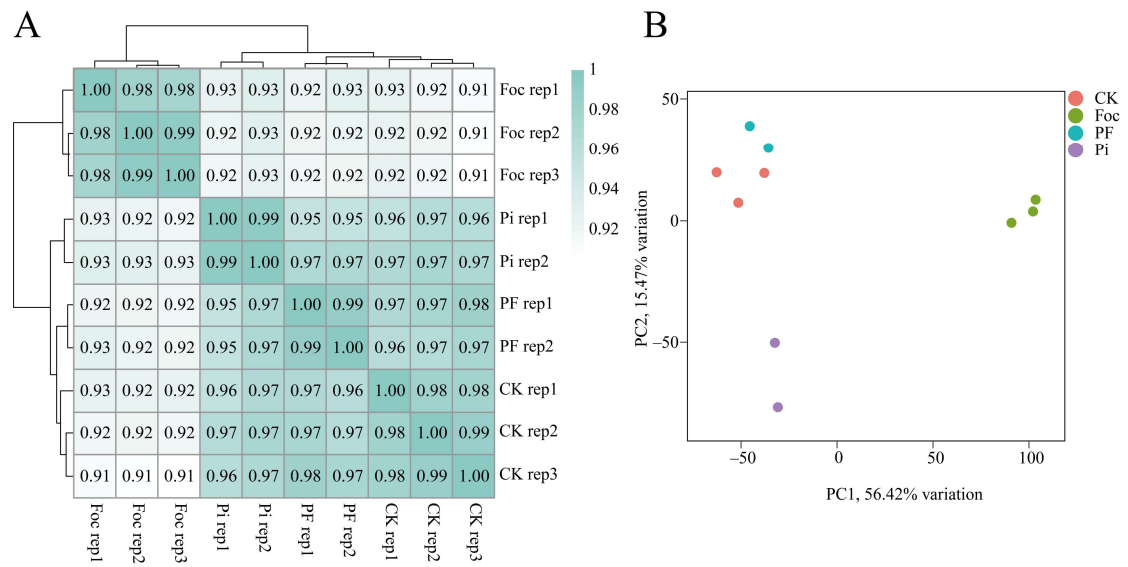

Figure S1. Heatmap (A) of Pearson correlation and PCA (B) among ten samples of RNA-seq.

Supplement: Supplementary file 1 [file ijms-25-12446-s001.zip › Fig.S1.pdf]

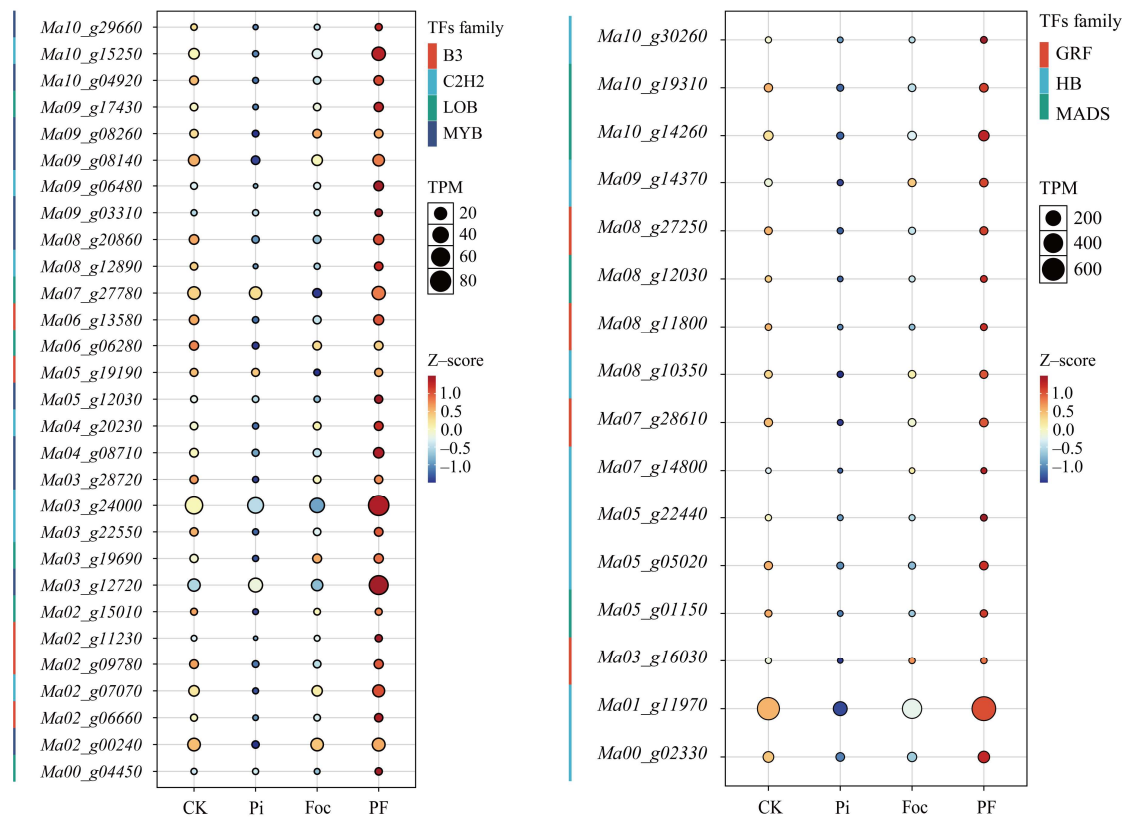

Figure S2. Bubble chart displaying expression of *B3*, *C2H2*, *LOB*, *MYB*, *GRF*, *HB*, *MADS* TF families in cluster 3.

Supplement: Supplementary file 1 [file ijms-25-12446-s001.zip › Fig.S2.pdf]

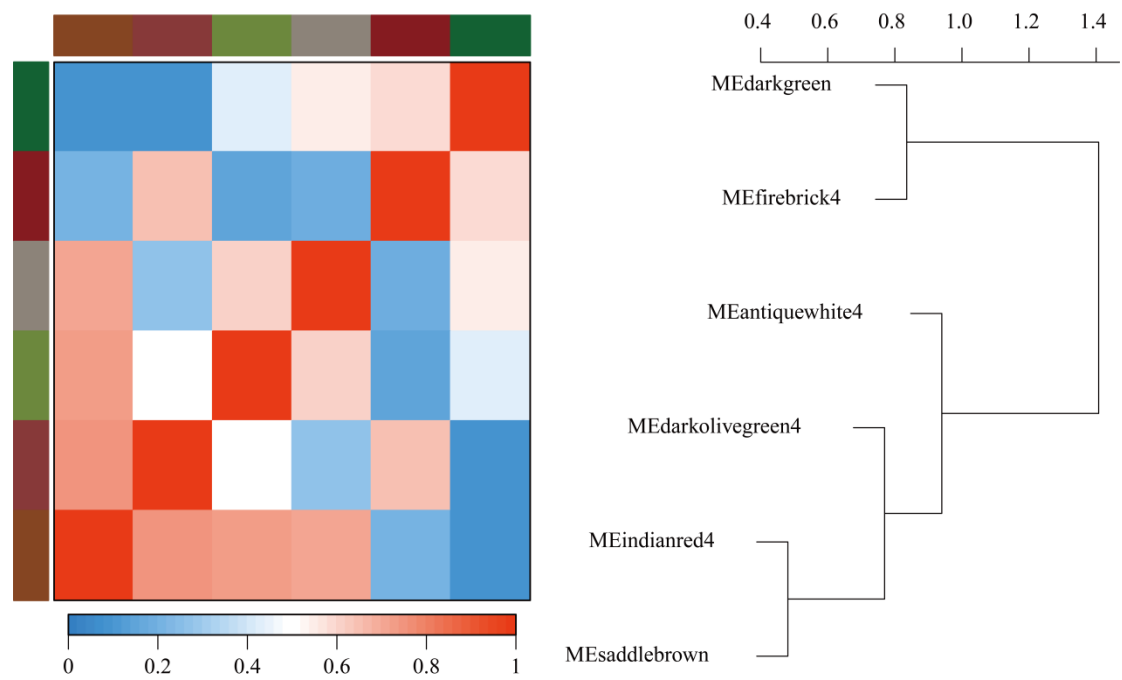

Figure S3. WGCNA eigengene dendrogram and eigengene adjacency heatmap.

Supplement: Supplementary file 1 [file ijms-25-12446-s001.zip › Fig.S3.pdf]

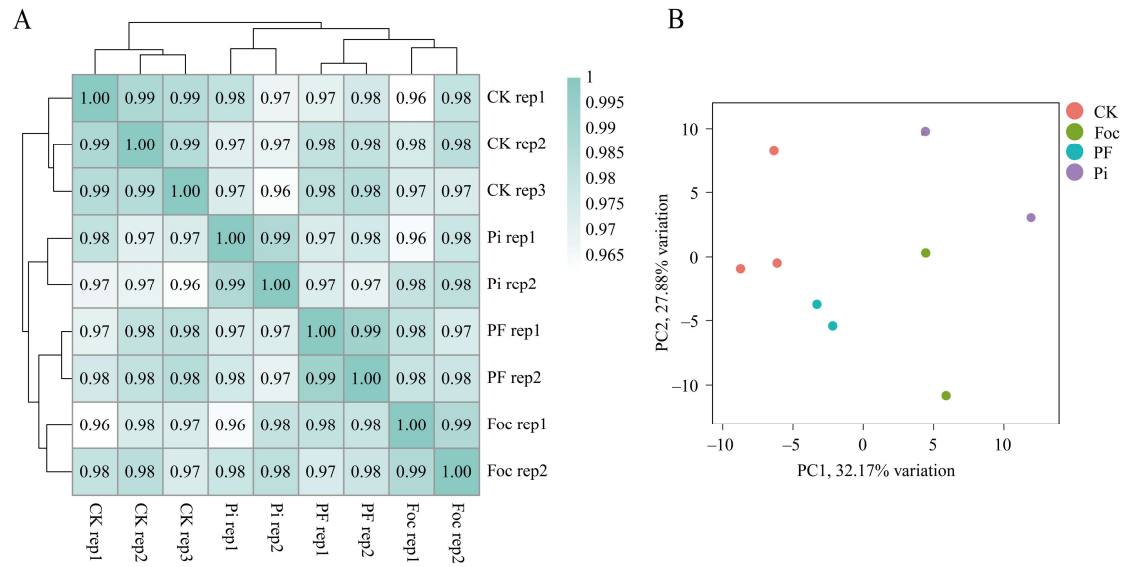

Figure S5. Heatmap (A) of Pearson correlation and PCA(B) among nine samples of sRNA.

Supplement: Supplementary file 1 [file ijms-25-12446-s001.zip › Fig.S5.pdf]

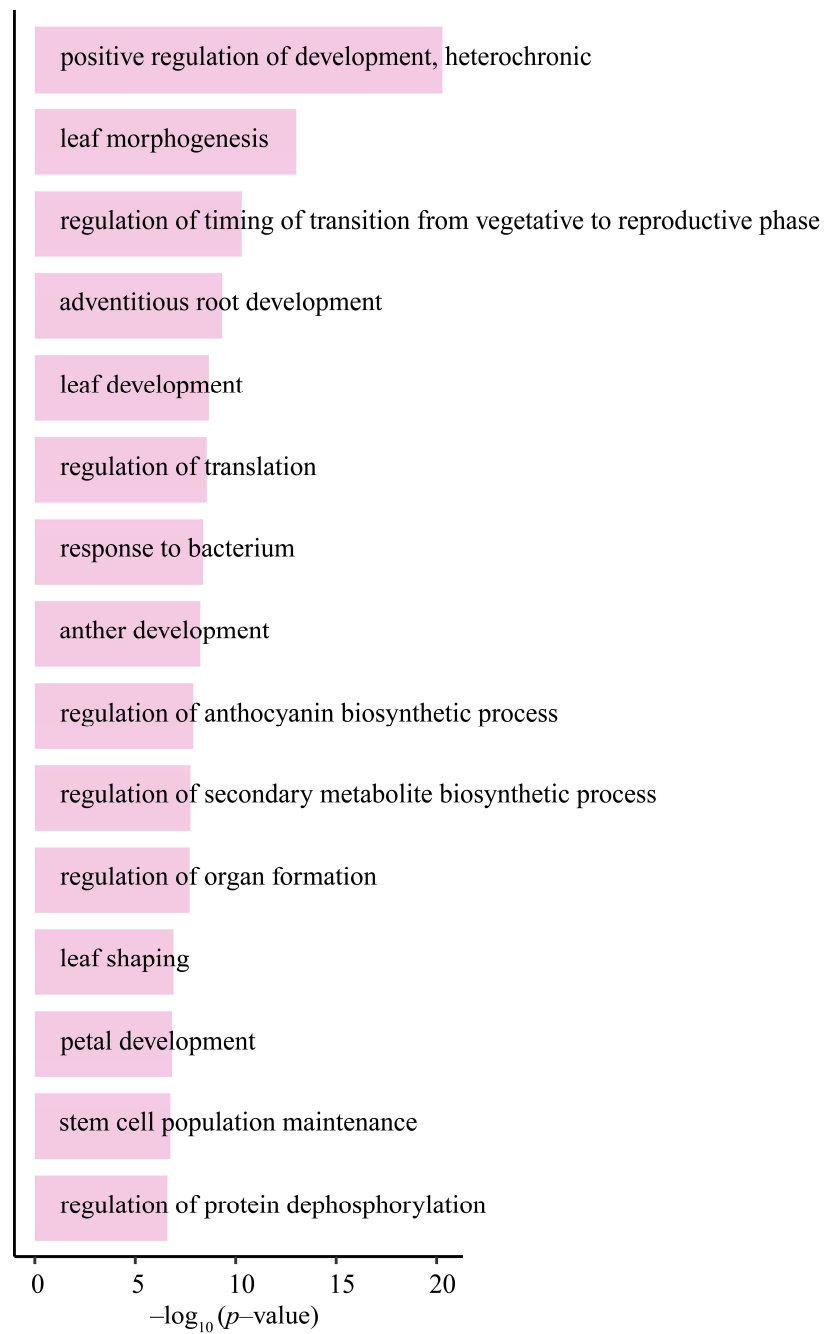

Figure S6. GO enrichment analysis of target genes of all miRNAs.

Supplement: Supplementary file 1 [file ijms-25-12446-s001.zip › Fig.S6.pdf]
